# An Optical Acoustic Detection System Based on Fabry Pérot Etalon Stability Structure

**DOI:** 10.3390/mi12121564

**Published:** 2021-12-16

**Authors:** Liyun Wu, Yongqiu Zheng, Chenyang Xue, Jiandong Bai, Jiamin Chen

**Affiliations:** 1 Key Laboratory of Instrumentation Science and Dynamic Measurement Ministry of Education, North University of China, Taiyuan 030051, China; wuliyun1211@126.com (L.W.); xuechenyang@nuc.edu.cn (C.X.); jdbai@nuc.edu.cn (J.B.); 18335160365@163.com (J.C.); 2 Taiyuan Institute of Technology, Taiyuan 030008, China

**Keywords:** stability structure, the Fabry Pérot Etalon (FPE), optical acoustic detection system, higher signal-to-noise ratio (SNR)

## Abstract

The optical acoustic detection system based on the Fabry Pérot Etalon (FPE) with high quality–factor (High Q) and stability structure is described and tested. The FPE contains two high–reflectivity Plano–Concave lenses, achieving high fineness and stability. The protective structure of the confocal stabilized FPE is composed of an invar tube, copper sheath, Bakelite sheath and aluminum housing to protect the sensor from the effects of ambient temperature and vibration. The audio signal is injected into the cavity through the sound hole located in the center of the cavity. Acoustic waves induce the vibration of the medium in the cavity, which leads to a simultaneous change in the FPE optical path and a shift of the interference spectrum. The acoustic detection system is built, and the frequency of the laser is locked on the resonant frequency points of the FPE by using phase modulation technology, so as to detect acoustic signals of different frequencies and amplitudes. In addition, the sensitivity of the proposed sensor exceeds 34.49 mV/Pa in the range of 20 Hz–20 kHz. A Signal-to-Noise Ratio (SNR) of 37 dB can be achieved at 20 Hz. Acoustic signal detection technology based on the FPE stability model is used to test the theoretical feasibility of the future high sensitivity Fabry Pérot Interferometric (FPI) acoustic sensors.

## 1. Introduction

Fabry Pérot Etalon (FPE) acoustic sensors have attracted increasing attention in the acoustic measurement field due to their advantages, namely their high sensitivity and compact structure [1,2,3]. The FPE sensors can be divided into the categories of high and low fineness, determined by the reflectivity of the cavity mirrors. Compared to the low fineness FPE sensor, the high fineness FPE sensors have much higher sensitivity, lower Total Harmonic Distortion (THD) [4] and highly reproduced acoustics signal in the audible frequency range (20 Hz–20 kHz).

Recently, a variety of Fabry Pérot (FP) acoustic sensors have been developed. According to the structure of FP, it can be divided into Fabry Pérot Interferometer (FPI) with variable cavity length and FPE with constant cavity length. FPI usually consists of a fiber end face [5,6,7,8] and a thin diaphragm [9,10]. Metal [11], polymers [12], graphene [13,14] and other materials [14,15] have been used to make diaphragms. The performance of the sensor is directly affected by the reflectivity of the fiber end face, the composition and geometric structure of the film material. At the same time, the reflectivity of the fiber end face is very low, only about 3%. Therefore, the fineness of FPI is very low, directly depending on the end face of the optical fiber [5] and the surface of the film on the end face of the optical fiber [9]. Coatings, special fibers and lenses have been used to improve the reflectivity of the fiber end face, but these methods are a difficult way to significantly increase the reflectivity of the cavity mirror and increase the manufacturing difficulty. In 2016, the Austrian XARION laser company developed a high-performance FPE acoustic sensor with a detection sensitivity of 80 mV/Pa and a dynamic range of 10 Hz–20 MHz [16,17,18]. However, the sensor head is composed of two flat mirrors, it is difficult for the light beam to couple with the FPE, the open cavity structure is greatly affected by the environment and the sound signal extraction is difficult. Therefore, a stable FPE structure is designed, which can well isolate the influence of the environment, and adopts two Plano–Concave lenses with high reflectivity to reduce the coupling difficulty and improve the fineness.

In this paper, the FPE acoustic sensor is tested, based on a stable structure, with high fineness and high sensitivity. In order to minimize the impact of ambient temperature and vibration on the sensor, the stable FPE is protected by four layers, namely an invar tube, copper sheath, Bakelite sheath and aluminum housing. At the same time, the FPE is formed by two Plano–Concave Lenses with high reflectivity. In addition, the sensitivity of the proposed sensor exceeds 34.49 mV/Pa in the range of 20 Hz–20 kHz, and achieves a Signal-to-Noise Ratio (SNR) of 37 dB at 20 Hz. The proposed sensor has other advantages, including an audible full-frequency acoustic detection system at around 20 Hz–20 kHz. For aural evaluation of the optical acoustic detection system, music recordings are made.

## 2. Sensor Design and Operation Principle

The FPE is a kind of optical cavity, which is composed of two Plano–Concave Spherical Lenses with coincident optical axes. A beam of light is reflected between the reflecting surfaces multiple times, resulting periodically in resonance curves in the optical frequency spectrum. For an ideal FPE, the transfer function *T*(*q*) is given by the Airy function [18,19]:

(1) $$T\left(q\right)=\frac{I_{t}}{I_{0}}=\frac{1}{1+\frac{4R\;\sin^{2}\;q/2}{{\left(1-R\right)}^{2}}}$$

where $I_{0\;}$ and $I_{r\;}$ is the intensity of incident and transmitted light, respectively. *R* is the reflectivity of the two interfaces; *q* is the phase difference in one round trip through etalon, which can be expressed as:

(2) q(n)=4πnd/λ

where *λ* represents the central wavelength of incident light; *d* represents the distances between the Plano–Concave lenses; *n* is the refractive index of the cavity medium. The finesse is used to measure the sharpness of transmitted light. It is defined as:

(3) $$F=\frac{\pi \sqrt{R}}{1-R}$$

The finesse depends solely on mirror reflectivity, as evident in the Formula (3). However, the finesse is an etalon whose performance is impaired due to diffraction losses, tilt angle or surface figure.

The key challenge in the design and manufacturing of FPE is to precisely control and retain the distance between the etalon mirrors, and ensure that the cavity length is unaffected by temperature, vibration or other factors. In order to achieve the high fineness of FPE, we need to select cavity mirrors with high reflectivity and an appropriate radius of curvature. The confocal etalon is a widely used stable FPE. In order to match the parameters of the laser, the radius of curvature of the FPE mirrors is 100 mm.

**Figure 1:**
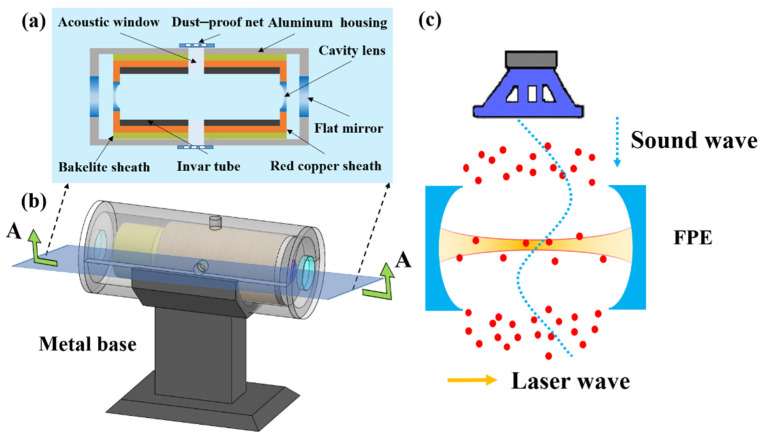
(**a**) The sectional view of the FPE on surface A–A; (**b**) the mechanical structure diagram of the FPE; (**c**) the detection principle of the acoustic detection structure. The sound wave (illustrated by the blue dotted line) constitutes the density change in the air medium between the cavity mirrors (represented by the red dots).

As shown in Figure 1a, the design of the cavity incorporates invar material (Nickel 36%, iron balance, carbon 0.02%, silicon 0.18%, manganese 0.35%) with a very small thermal expansion coefficient (9 × 10^−7^ m/°C at −250 °C to 200 °C) [20], which slows down the temperature change of the cavity. The cavity mirrors are attached to both ends of the invar tube. In order to reduce the effect of ambient temperature on the length of the cavity, the outer layer of the invar tube is lined with a layer of red copper material with good thermal conductivity. The outer layer of red copper is a Bakelite sheath, which has a shockproof function and also helps to preserve heat. The whole cavity is fixed in an aluminum housing with two light openings, sealed by two flat mirrors. This design and manufacture ensure that the FPE has strong stability, and effective protection against external factors such as disturbance of the external airflow, mechanical vibration and environmental temperature change [20]. In order to enable detection of the sound signal, the acoustic windows are opened on the left side, right side and upper side of the cavity. The diameter of the acoustic windows is 10 mm and penetrates through four layers of protective structure. The acoustic windows are covered by dust-proof nets, as shown in Figure 1a. FPE designed and processed in this way has strong stability, and has a good protective effect on external factors such as external air flow disturbance, mechanical vibration and environmental temperature changes.

When an acoustic signal is applied to the FPE, it causes the refractive index of the medium to change. The optical path length is changed by the change in the refractive index. This leads to the resonance spectrum to drift, which can be detected by monitoring the light intensity transmitted by the resonator. The detection principle of the FPE for acoustic sensing is shown in Figure 1c.

**Figure 2:**
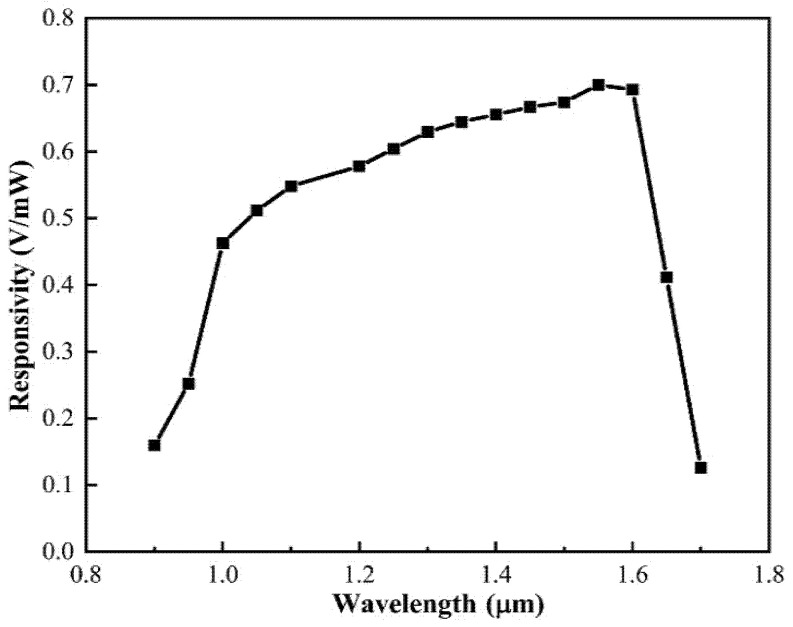
Typical responsivities of the model 2053.

Therefore, the voltage sensitivity $S_{V}$ can be written as:

(4) $$S_{V}=\frac{dV_{o}}{dP}=\frac{dV_{o}}{dp_{t}}\cdot \frac{dp_{t}}{dI_{t}}\cdot \frac{dI_{t}}{dn}\cdot \frac{dn}{dP}$$

where $V_{o}$ is the system output voltage, *P* is acoustic pressure, *n* is the refractive index of the medium, $I_{t}$ is the transmitted optical power of the FPE and $p_{t}$ is the input power of the photo detector in Watts. It can be seen from the photo detector (New Focus 2053-FS) that the relationship between $V_{o}$ and $I_{t}$ can be expressed as

(5) $$\frac{dV_{o}}{dp_{t}}=ℜ\cdot G$$

where *G* is the amplifier’s gain setting, ℜ is the photo detector’s response factor in V/mW, as seen in Figure 2, and the value of ℜ at 1550 nm is 0.7 V/mW [21].

(6) $$\frac{dp_{t}}{dI_{t}}=S$$

where *S* is the Active Area of the model 2053-FS in 0.08 mm^2^.

The intensity transmitted from an FPE is the product of the input intensity $I_{o}$ and a transfer function *T*(*q*), which for an ideal resonator, is given by the Airy function. Thus, the refractive index sensitivity $S_{n}$ is;

(7) $$S_{n}=\frac{dI_{t}}{dn}=\frac{-1}{{\left(1+\frac{4R\;\sin^{2}\;q/2}{{\left(1-R\right)}^{2}}\right)}^{2}}\cdot \frac{4R}{{\left(1-R\right)}^{2}}\cdot \frac{2\pi d\sin q}{\lambda }I_{0}$$

The Lorentz–Lorentz formula relates to the macroscopic optical properties of the medium [22].

(8) $$\alpha =\frac{3}{4\pi N}\frac{n^{2}-1}{n^{2}+2}$$

where *n* is the refractive index of the medium; *N* represents the number density of molecules; α is a coefficient, which is determined by the mean polarizability of isotropic molecules. An acoustic process is adiabatic, that is, a fast process without heat exchange. Based on the adiabatic state equation and Clapeyron equation of an ideal gas with a certain mass, the motion equation, continuity equation and state equation of acoustic waves, the refractive index in an ideal gas can therefore be expressed as:

(9) $$\frac{T^{\gamma }}{P^{\gamma -1}}=const$$

(10) $$dn\approx \frac{3\alpha }{2\kappa_{B}\gamma T}dP$$

(11) $$\left(n-1\right)\times 10^{6}=\left(\frac{273.15}{1013.25}\cdot \frac{p}{T}\cdot \left(287.6155+\frac{1.62887}{\lambda^{2}}+\frac{0.01360}{\lambda^{4}}\right)\right)-11.27\cdot \frac{e}{T}$$

(12) $$e=RH\cdot 610.78\cdot \exp\left(17.269\cdot \left(T-273.16\right)/\left(T-35.86\right)\right)\begin{array}{cc} & T>273.15 \end{array}$$

where *T* is the absolute temperature; the unit is K; *P* is the pressure of the gas; the unit is hPa; $\kappa_{B}$ is the Boltzmann constant; *γ* is the specific heat ratio of gas; *λ* is the laser wavelength of μm; RH is the relative humidity and *e* is the intermediate parameter [23].

**Figure 3:**
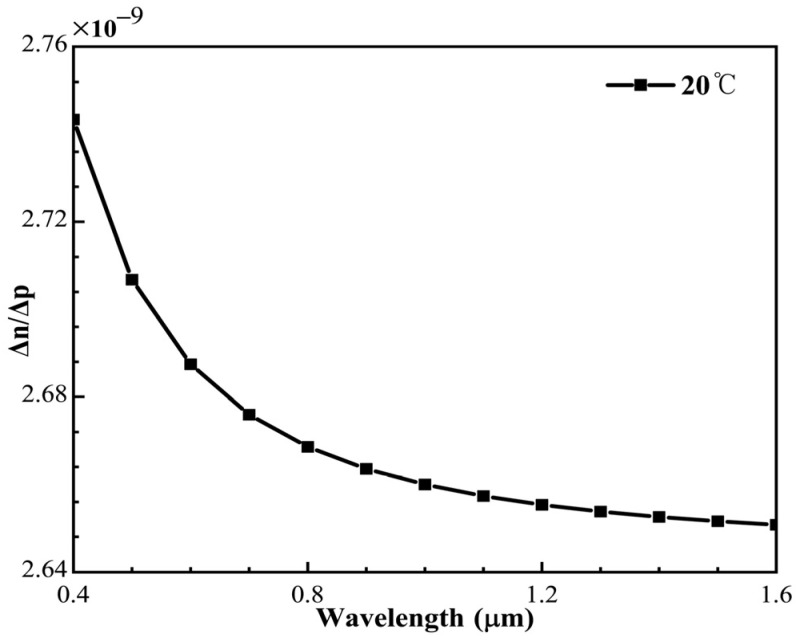
The change in refractive index is caused by unit sound pressure at different light wavelengths.

From Formula (10), we can see that the change in refractive index in an ideal gas is a linear function of the change in sound pressure. According to the Edlen formula of Formula (11), at 20 °C, the central wavelength of 1550 nm, and *RH* is 0.5, $∆n\approx ∆p\cdot 2.6511\times 10^{-9}$, as shown in the Figure 3.

Therefore, the phase sensitivity $S_{\phi }$ is:

(13) $$S_{\phi }=\frac{d\phi }{dP}=\frac{d\phi }{dn}\cdot \frac{dn}{dP}=\frac{4\pi d}{\lambda }\cdot \frac{S_{V}}{ℜGSS_{n}}$$

## 3. Acoustic Measurement and Discussion

**Figure 4:**
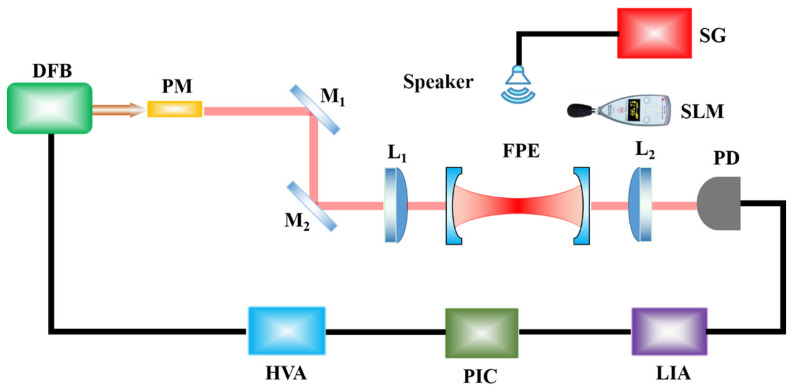
Schematic diagram of the acoustic detection system. DFB, Distributed Feedback laser; PM, Phase Modulator; M_1,2_, Plane Mirrors; L_1,2_, Plano–Convex Lenses; PD, Photoelectric Detector; LIA, Lock-In Amplifier; PIC, Proportional plus Integral Controller; HVA, High Voltage Amplifier; SG, Signal Generator; SLM, Sound Level Meter.

The experimental setup for acoustic sensing is depicted in Figure 4. A Distributed Feedback (DFB, the central wavelength of 1550 nm, NKT E15) laser with the Full Width at Half Maximum (FWHM) of 1 kHz serves as the light source. Two pieces of 45° total reflector with a radius of 12.7 mm and a thickness of 3 μm (M_1_, M_2_, BB0511-E04, Thorlabs, Newton, NJ, USA) are attached to collimate the optical path. The FPE is placed in the middle of two Plano–Convex lenses with a focal length of 100 mm (L_1_, L_2_, LC1975-C, Thorlabs), with the convex surface facing the FPE. The Newport 2053 Photoelectric Detector (PD) is used to convert optical signals into electrical signals. The signal is inputted into the digital Lock-In Amplifier (LIA, Stanford Research Systems, SR844, bandwidth 25 kHz–200 MHz), and the error signal is generated after mixing and low-pass filtering. The control signal is obtained by a Proportional plus Integral Controller (PIC). The control signal is amplified by a High Voltage Amplifier (HVA, Shandayuguang, Taiyuan, China) and applied to the laser, so that the working frequency of the laser can operate stably at the resonant frequency of FPE [24].

The loudspeaker excited by the Signal Generator (SG, AFG31000, Tektronix, Beaverton, OR, USA) is used as the acoustic source, which can generate sinusoidal acoustic waves in a specific frequency and amplitude. A handheld Sound Level Meter (SLM, AWA5661) is used for calibrating the sound pressure.

**Figure 5:**
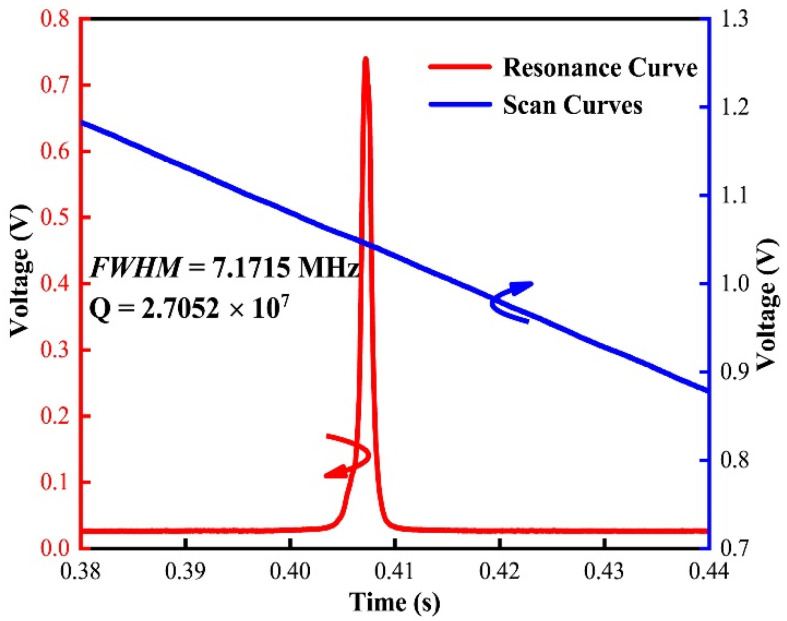
The test results of the proposed signal detection system. The blue line is the laser scanning curve; the red line is the spectral curve.

The performance of the proposed sensor is tested, as shown in Figure 5; the FWHM of the proposed sensor is 7.1715 MHz, which can be obtained according to the voltage difference of the laser scanning curve at the FWHM of the resonance curve [25].

**Figure 6:**
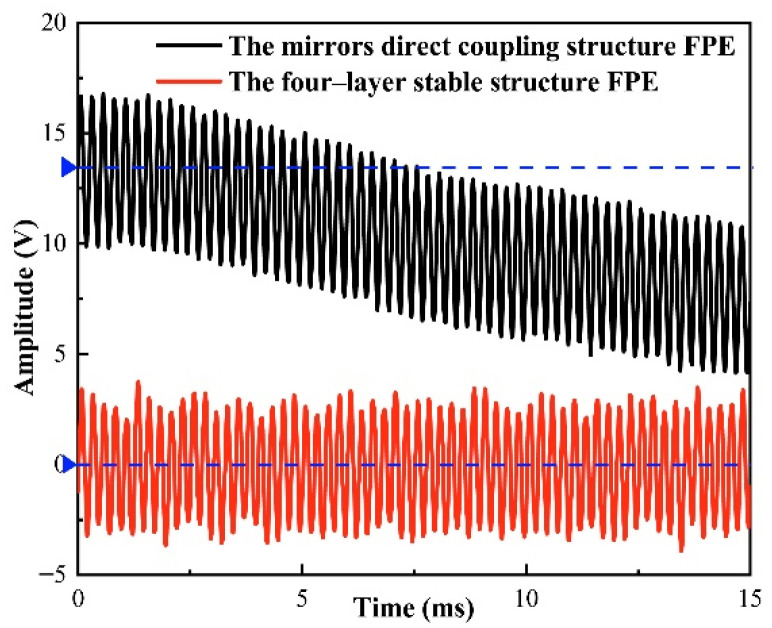
The influence of environment on the structure of mirrors directly coupled FPE and the four-layer stable FPE.

It can be seen from Figure 6 that under the same ambient temperature and vibration conditions, the acoustic signal detection system based on the four-layer stable structure FPE designed by us has not deviated from the center line. However, based on the mirrors direct coupling structure FPE, the detected acoustic signal deviates from the center line due to external environments such as temperature and vibration. It can be seen that the structure we designed can effectively reduce the influence of ambient temperature and vibration on the detection of acoustic signals.

**Figure 7:**
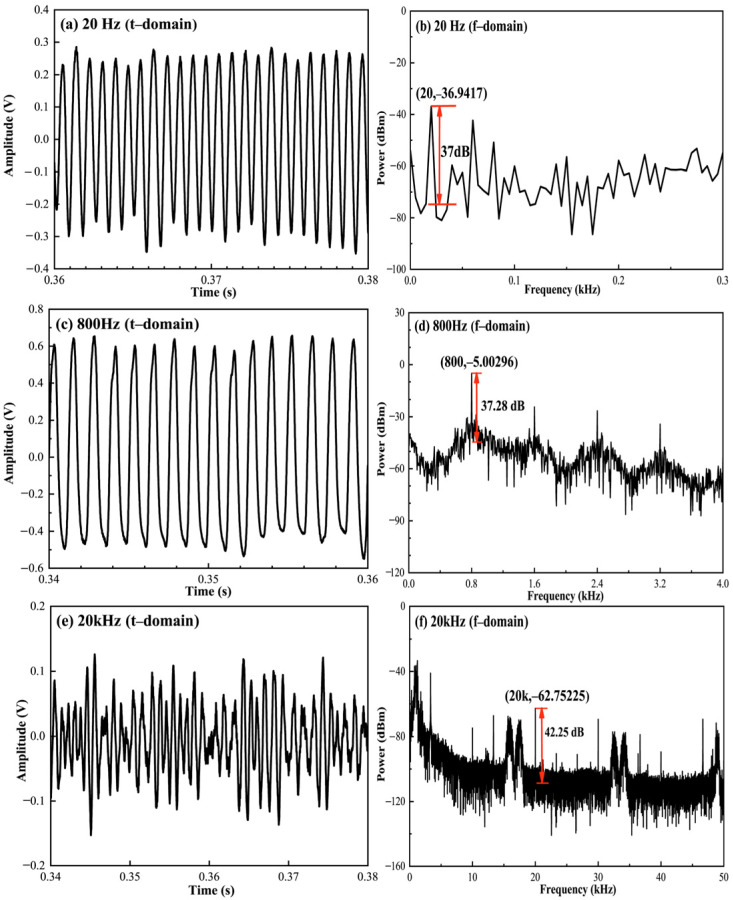
Outputs of the acoustic detection system at (**a**) 20 Hz; (**c**) 800 Hz; (**e**) 20 kHz and their corresponding frequency spectral (**b**,**d**,**f**).

Therefore, the FWHM and Q value are 7.1715 MHz and 2.7052 × 10^7^. Figure 7 shows the response results of the proposed sensor in both time and frequency domains at 20, 800 and 20 kHz. When an acoustic pressure of 12.6 mPa (corresponding to 56 dB Sound Pressure Level (SPL) re 20 μPa) is applied to the sensor head in the frequency range of 20 Hz–20 kHz, the sinusoidal acoustic signal is detected with no observable distortion. A SNR of 37 dB, with the noise floor approximately −74 dB for a 5 Hz resolution bandwidth, can be observed in Figure 7b. The minimum detectable pressure of the corresponding noise limit is 7.97 Pa/Hz^1/2^. The harmonics shown in Figure 7b,d allow us to clearly identify the detected acoustic frequency, demonstrating that our proposed sensor would effectively identify external acoustic interference.

**Figure 8:**
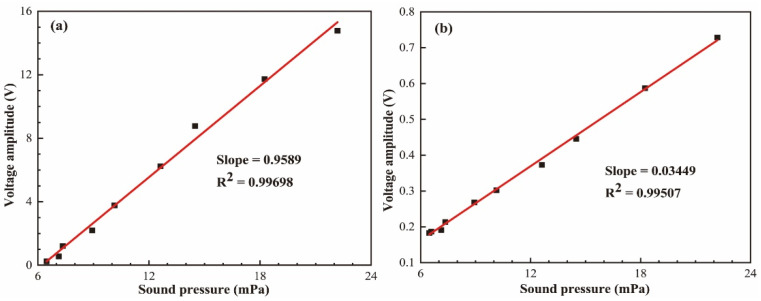
The output signals of the proposed sensors, varying the applied acoustic pressure, at frequencies of (**a**) 20 Hz and (**b**) 20 kHz.

Figure 8 shows the output signals of the proposed sensor at 20 Hz and 20 kHz when the applied acoustic pressure is increased from 6.472 to 22.183 mPa (corresponding to 50.2 db–60.9 dB sound pressure level re 20 μPa) by controlling the signal generator voltage. This suggests our proposed acoustic sensor shows an excellent linear response with an R-squared value of 0.99. In the experiment, the minimum detectable acoustic pressure was found to be 54 dB, limited by the environmentally acoustic noise (−50 dB). The value can be decreased by putting the sensor head in a silent environment such as an acoustic isolation box. The slope of this curve indicates that the dynamic response has a sensitivity of 0.9589 V/Pa at 20 Hz and 0.03449 V/Pa at 20 kHz.

**Figure 9:**
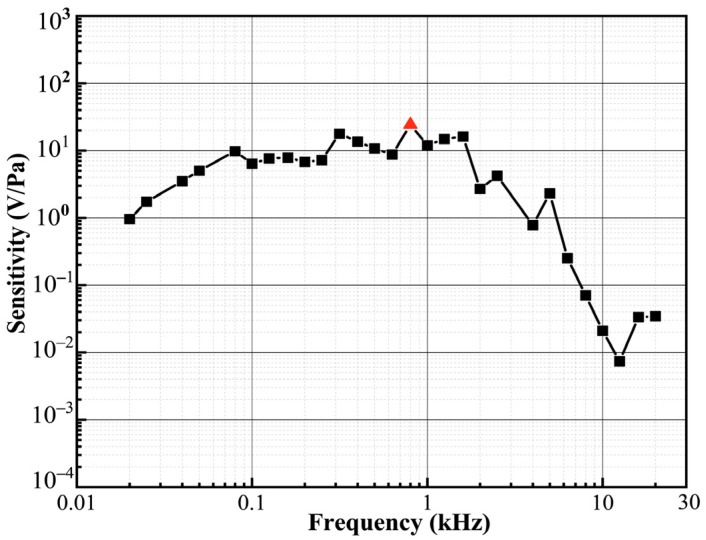
Frequency response of the acoustic detection system.

The performance of the acoustic signal detection system was tested according to ISO standards of 266–1975. Figure 9 shows the frequency response of the proposed sensor ranging from 20 Hz to 20 kHz, which exhibits a dominant resonance peak at approximately 800 Hz.

**Figure 10:**
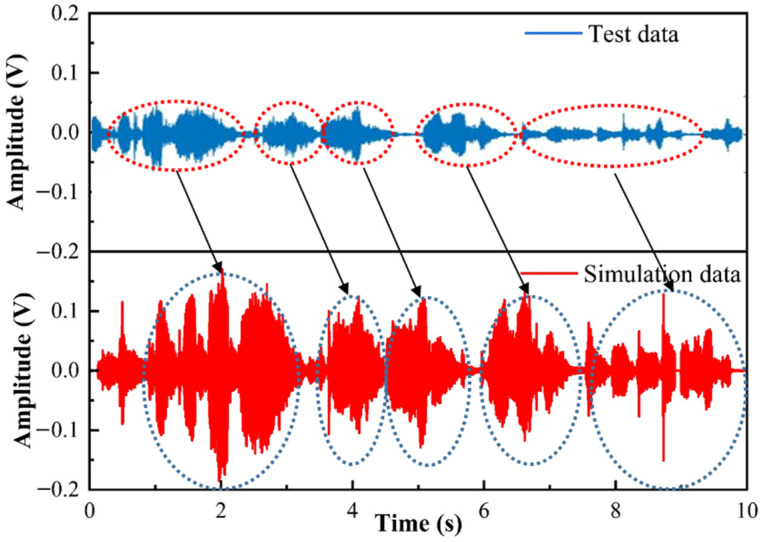
Comparison of simulation data and test data.

For an auditory evaluation of the acoustic detection system, music recordings are made. As shown in Figure 10, the recording data of the sensor are compared with the simulation data by the MATLAB software. In the experiment, the recording duration is 10 s, the sampling rate is 10 k and the data points are 100 k. The detected sound signal can correspond to each note of the simulated sound signal, and the high-pitched characters can be clearly displayed, but the sound amplitude is different, and the detected signal has noise and partial distortion because the data collected by the proposed sensor are affected by the operating noise of instruments, photoelectric noise and other noise. However, the simulation data was collected through a computer microphone and processed by Matlab software, and computer operating noise had an impact on the simulation data [26].

We compared the proposed acoustic sensor detection system with other sensors with different principles and structures in recent years, as shown in Table 1. Compared with other sensors, the acoustic detection system of this structure has the highest sensitivity, although it is larger than the other sensors. At the same time, the experimental conditions limit the detection of the frequency response range. In addition, the other advantage of our structure is that it can be used for acoustic signal detection in harsh environments.

## 4. Conclusions

In summary, a high-sensitivity and high-fineness acoustic sensor based on FPE was used. The change in refractive index, caused by the acoustic pressure, resulted in the optical path difference variation. For the stable optical structure and testing principle, the proposed sensor has a high sensitivity of 0.9589 V/Pa and SNR of 37 dB at 20 Hz. At the same time, the proposed sensor exhibits good linearity with an R-squared value of 0.99698 when the applied acoustic pressure is increased from 50.2 to 60.9 dB. The sensor achieved a sensitivity of 24 V/Pa at the audible frequency range (20 Hz–20 kHz).

## Figures and Tables

**Table 1:** The Performance of sensors based on different principles and structures.

Type	Testing Principle	Sensitivity (mV/Pa)	SNR (dB)	Frequency Response Range (Hz)	Size (mm × mm)	Ref.
B&K 4189	Changes in capacitance	50		6.3–20 k	12.7 × 11.7	[27]
Xarion Eta 100 Ultra	Changes in refractive index	0.35	52	10–1 M	5 × 33	[28]
FPI	Vibration of thin membrane	57.3	30	1–20 k	2.5 × 9	[29]
Proposed	Changes in refractive index	958.9	42.25	20–20 k ^1^	80 × 100	

^1^ Limitation of experimental conditions.

## References

[1] Liu B., Lin J., Wang J., Ye C., Jin P. (2016). MEMS-based high-sensitivity Fabry–Perot acoustic sensor with a 45° angled fiber. IEEE Photonics Technol. Lett..

[2] Zheng H., Lv R., Zhao Y., Wang X., Lin Z., Zhou Y., Zhao Q. (2021). A novel high accuracy optical path difference compensation method based on phase difference technology. Opt. Lasers Eng..

[3] Chen J., Xue C., Zheng Y., Wu L., Chen C., Han Y. (2021). Micro-fiber-optic acoustic sensor based on high-Q resonance effect using Fabry-Pérot etalon. Opt. Express.

[4] Bing Y., Anbo W., Pickrell G.R. (2006). Analysis of Fiber Fabry–Pérot Interferometric Sensors Using Low-Coherence Light Sources. J. Lightwave Technol..

[5] Zhang W., Li H., Zhu L., Dong M., Meng F. (2021). Dual-parameter optical fiber probe based on a three-beam fabry-perot interferometer. IEEE Sens. J..

[6] Rodríguez-Quiroz O., Domínguez-Flores C.E., Monzón-Hernández D., Morales-Narvaez E., Minkovich V.P., López-Cortés D. (2020). Unambiguous refractive-index measurement in a wide dynamic-range using a hybrid fiber Fabry-Perot interferometer assisted by a fiber Bragg grating. Opt. Laser Technol..

[7] Yang X., Wu S., Cheng H., Ma J., Wang S., Liu S., Lu P. (2021). Simplified highly-sensitive gas pressure sensor based on harmonic Vernier effect. Opt. Laser Technol..

[8] Chen D., Qian J., Liu J., Chen B., An G., Hong Y., Jia P., Xiong J. (2020). An in-line Fiber optic fabry–Perot sensor for high-temperature vibration measurement. Micromachines.

[9] Zhang W., Lu P., Ni W., Xiong W., Liu D., Zhang J. (2020). Gold-diaphragm based Fabry-Perot ultrasonic sensor for partial discharge detection and localization. IEEE Photonics J..

[10] Yang S., Feng Z., Jia X., Pickrell G., Ng W., Wang A., Zhu Y. (2020). All-sapphire miniature optical fiber tip sensor for high temperature measurement. J. Lightwave Technol..

[11] Wang S., Lu P., Liu L., Liao H., Sun Y., Ni W., Fu X., Jiang X., Liu D., Zhang J. (2016). An infrasound sensor based on extrinsic fiber-optic Fabry–Perot interferometer structure. IEEE Photonics Technol. Lett..

[12] Wang Q., Yu Q. (2010). Polymer diaphragm based sensitive fiber optic Fabry-Perot acoustic sensor. Chin. Opt. Lett..

[13] Ni W., Lu P., Fu X., Zhang W., Shum P.P., Sun H., Yang C., Liu D., Zhang J. (2018). Ultrathin graphene diaphragm-based extrinsic Fabry-Perot interferometer for ultra-wideband fiber optic acoustic sensing. Opt. Express.

[14] Zhang W., Lu P., Qu Z., Fan P., Sima C., Liu D., Zhang J. (2021). High sensitivity and high stability dual Fabry-Perot interferometric fiber-optic acoustic sensor based on sandwich-structure composite diaphragm. IEEE Photonics J..

[15] Lyu D., Peng J., Huang Q., Zheng W., Xiong L., Yang M. (2021). Radiation-resistant optical fiber Fabry-Perot interferometer used for high-temperature sensing. IEEE Sens. J..

[16] Fischer B. (2016). Optical microphone hears ultrasound. Nat. Photonics.

[17] Fischer B., Sarasini F., Tirillò J., Touchard F., Chocinski-Arnault L., Mellier D., Panzer N., Sommerhuber R., Russo P., Papa I. (2019). Impact damage assessment in biocomposites by micro-CT and innovative air-coupled detection of laser-generated ultrasound. Compos. Struct..

[18] Preisser S., Rohringer W., Liu M., Kollmann C., Zotter S., Fischer B., Drexler W. (2016). All-optical highly sensitive akinetic sensor for ultrasound detection and photoacoustic imaging. Biomed. Opt. Express.

[19] Chen M., Xie S., Zhou G., Wei D., Wu H., Takahashi S., Matsumoto H., Takamasu K. (2019). Absolute distance measurement based on spectral interferometer using the effect of the FSR of a Fabry–Perot etalon. Opt. Lasers Eng..

[20] Zhu S., Yongrui G., Minzhi X., Huadong L. (2018). The design and realization of high fineness passive optical resonator and its application in lasers’ frequency stability. J. Quantum Opt..

[21] 2051-2053-User-Manual-RevB. https://www.newport.com.cn/p/2053-FS-M.

[22] Ciddor P.E. (2002). Refractive index of air: 3. The roles of CO2, H2O, and refractivity virials. Appl. Opt..

[23] Rüeger J.M. Refractive indices of light, infrared and radio waves in the atmosphere. Report of the Ad-Hoc working party of the IAG special commission SC3 on fundamental parameters (SCFC), 1999–2003. Proceedings of the 23rd General Assembly of IUGG.

[24] Dass S., Chatterjee K., Kachhap S., Jha R. (2021). In reflection metal coated diaphragm microphone using PCF modal interferometer. J. Lightwave Technol..

[25] Chen J., Zheng Y., Xue C., Zhang C., Chen Y. (2018). Filtering effect of SiO2 optical waveguide ring resonator applied to optoelectronic oscillator. Opt. Express.

[26] Hu H., Chou H., Lee T. (2021). Robust blind speech watermarking via fft-based perceptual vector norm modulation with frame self-synchronization. IEEE Access.

[27] TYPE 4189 Free-Field Microphone. https://www.bksv.com/en/transducers/acoustic/microphones/microphone-cartridges/4189.

[28] Eta100 Ultra Optical Microphone. https://xarion.com/en/products/eta100-ultra.

[29] Liu L., Lu P., Wang S., Fu X., Sun Y., Liu D., Zhang J., Xu H., Yao Q. (2016). UV Adhesive diaphragm-based FPI sensor for very-low-frequency acoustic sensing. IEEE Photonics J..

